# Betting is Loving and Bettors are Predators: A Conceptual Metaphor Approach to Online Sports Betting Advertising

**DOI:** 10.1007/s10899-017-9727-x

**Published:** 2017-10-30

**Authors:** Hibai Lopez-Gonzalez, Frederic Guerrero-Solé, Ana Estévez, Mark Griffiths

**Affiliations:** 1 International Gaming Research Unit, Psychology Division, Nottingham Trent University, 50 Shakespeare Street, Nottingham, NG1 4FQ UK; 2 Department of Communication, Pompeu Fabra University, Roc Boronat, 138, 52.825, 08018 Barcelona, Spain; 3 Psychology Department, University of Deusto, Apartado 1, 48080 Bilbao, Spain

**Keywords:** Sports betting, Advertising, Soccer, Online, Metaphor, Narrative, Gambling

## Abstract

The legalisation of online gambling in multiple territories has caused a growth in the exposure of consumers to online sports betting (OSB) advertising. While some efforts have been made to understand the visible structure of betting promotional messages, little is known about the latent components of OSB advertisements. The present study sought to address this issue by examining the metaphorical conceptualisation of OSB advertising. A sample of Spanish and British television OSB advertisements from 2014 to 2016 was analysed (N = 133). Following Lakoff and Johnson’s conceptual metaphor theory, four main structural metaphors that shaped how OSB advertising can be understood were identified: betting as (1) an act of love, (2) a market, (3) a sport, and (4) a natural environment. In general, these metaphors, which were found widely across 29 different betting brands, facilitated the perception of bettors as active players, with an executive role in the sport events bet upon, and greater control over bet outcomes.

## Introduction

Online sports betting (OSB) is a globally growing economy. For instance, in 2016 in Europe, OSB represented an annual business of €16.5bn in gaming gross revenue (European Gaming and Betting Association 2016). The normalisation of OSB has run in parallel to its gradual legalisation in many countries since the mid-2000s. The legal status of OSB in the European Union has led to a large and increasing number of betting operators being legally available to consumers. Consequently, this has led to much increased competition between brands to position themselves and attract customers in a relatively new market (European Commission 2012). Unsurprisingly, OSB brands have resorted to advertising as one of the most efficient tools to increase their market share and competitive edge. Advertising expenditure continued to grow during the financial crisis even in mature markets like the UK (Davies 2016) while simultaneously, society has become more concerned about the unknown psychosocial impact that ubiquitous and constant exposure to gambling advertising might have on vulnerable groups (Gainsbury et al. 2013; McMullan et al. 2012).

The causal mechanisms of advertising influence on gambling behaviour remain largely unknown despite the growing body of scientific evidence in the field (Griffiths 2005). A critical review of gambling advertising research concluded that while the impact of advertising on problem gambling development was arguably small, it was unlikely that gambling advertising had no impact whatsoever on it (Binde 2014). In correlational studies, problems gamblers have typically self-reported greater exposure to gambling advertising (Hing et al. 2014) but whether the problem incentivised the bigger exposure or vice versa remains unclear, although more recent work has indeed argued a causal path between gambling advertising exposure, and problem gambling (Hanss et al. 2015).

OSB as a distinctive form of gambling has not received much empirical attention until very recently. Studies have shown that characters in betting advertisements usually focus on online platforms to bet, portraying characters in the narratives as predominantly male, sophisticated, and tech-savvy. This profile aligns with that of the target audience envisaged by the OSB operators (Gassmann et al. 2017; Hing et al. 2016). Betting advertising arguably conducts a dual strategy. On the one hand, advertisements accentuate the safety of betting with the brand; studies have shown that 47% of the adverts contain claims about free money or money back (Hing et al. 2017). On the other hand, advertising regularly promotes multi-bet and exotic bet scenarios in which the expected loss is higher for the bettor (Gainsbury and Russell 2015). The promotion of so-called ‘risk-free’ bets has been subject to criticism and a number scholars have argued against its communication without limitations (Hing et al. 2017; Sproston et al. 2015; Thomas et al. 2015). In this sense, the use of familiar faces and celebrities in betting advertisements has been theorised as having a perceived risk-lowering effect in bettor’s minds, given the transference of trustworthiness, credibility, and success that sport stars attach to the betting brand (Lamont et al. 2016).

Exposure to betting advertising of sport fans during televised sport is commonly held to be widespread across many different countries (Hing et al. 2017). National Rugby League (NRL) fans in Australia have been reported to watch over 15 min of gambling advertising per game on average (Gordon and Chapman 2014). Other research reported there were up to 322 ‘episodes of marketing’ in three NRL games, including 247 visual brand impacts from electronic banners around the pitch (Lindsay et al. 2013). Such penetration and extent of betting advertising is a likely contributory factor in strengthening the mental association between sport and gambling. Related to this, children aged 5–12 years in an Australian experiment mistakenly recalled betting brands sponsoring sport teams even when those brands were not the actual sponsors (Bestman et al. 2015). Furthermore, betting advertising analysis has demonstrated that narratives encourage consumers to think about the myth of gambling as a sport and to empathise with the characters (Milner et al. 2013). Other attributes found to increase the perception of bettors regarding their probability of winning include authoritative voice-overs, referring to bettors as clients (i.e., betting is a business), and the use of integrated banners in the narration (Milner et al. 2013).

The multiplicity of OSB advertising incarnations has made assessments of its impact more complex. Sport content consumers receive advertising stimuli from many platforms, including stadium banners, electronic banners around the field, shirt sponsors, team official partnerships, sport news, and pop-up messages, as well as traditional radio and television advertising (Thomas et al. 2012a). In addition, OSB is now beginning to integrate with the digital, gambling, and sport spheres, converging progressively with adjacent industries such as poker, trading, and social gaming, as well as sport and data journalism (Lopez-Gonzalez and Griffiths 2016a). Given this wide array of advertising platforms and formats, many studies have focused on external, format-specific, attributes of OSB advertising messages and their influence on betting behaviour (Hing et al. 2014, 2017; Lamont et al. 2011; Milner et al. 2013; Sproston et al. 2015; Thomas et al. 2015), while less work has been devoted to the latent attributes of such messages (Gainsbury et al. 2016).

The present study sought to address the paucity of knowledge concerning the internal structure of OSB advertising by analysing the metaphorical core of its messages. The underlying assumption in the present paper is that OSB advertisements, irrespective of brand specificity, construct their messages around a few commonly shared metaphors that shape the way bettors think about their betting. Drawing on Lakoff and Johnson’s (1980) Conceptual metaphor theory (CMT), the present paper examines the structural metaphors underpinning OSB advertising and the consequences for bettors of such characterisation.

## Conceptual Metaphor Theory

Heavily influenced by cognitive linguistics, Lakoff and Johnson defined a metaphor as ‘understanding and experiencing one kind of thing in terms of another’ (Lakoff and Johnson 1980, p. 5). This brief definition does not consider metaphors as tropes of figurative language, sorts of rhetoric, or poetic elements used to embellish literature. Conversely, in CMT, metaphors are linguistic manifestations of conceptual thinking. If words are shaped in the form of metaphors in everyday language it is because the concepts behind those words are also shaped metaphorically in people’s minds. Therefore, ordinary language is permeated with metaphors that speakers do not notice or recognise as such in conversational situations. In Lakoff and Johnson’s formulation, ordinary language is relevant because the authors conceive it as a materialisation and symbol of conceptual thinking, or in Schmitt’s words, an ‘homology of speech and thought’ (2005).

Embodied in the CMT definition is the idea of a target domain (*one kind of thing*) and a source domain (*in terms of another*). The target borrows meaning from the source, and in doing so it becomes structured by it. Lakoff offers a number of examples from English to make their case. For instance, in the ‘Time is Money’ metaphor, time (target) is understood in terms of money (source). Without necessarily noticing, English speakers (and other speakers in this case, due to the universality of this metaphor) refer to time using expressions like *wasting* time, *saving* time, *investing* time, *running out* of time, *putting aside* some time, or time being *worth* your while (Lakoff and Johnson 1980). Similarly, in the metaphor ‘Theory is Building’, speakers conceptualise theories as concepts with *foundations* that must be *constructed* or *built* from bottom to top, that need *support* not to *fall apart*. *Shaky* theories can *collapse* while *strong* theories do not. Lakoff and Johnson refer to these specific forms and argue that they are systematically built as ‘structural metaphors’ (1980).

The influence of CMT on scholarly work has become evident in recent years. CMT has produced or indirectly influenced research on heterogeneous topics including the public shaping of the gun debate in the US (David et al. 2016), the negative impact of metaphors representing cancer as an enemy that patients need to fight against (Hauser and Schwarz 2015), the use of metaphors to communicate to the public complex issues like gene therapy (Nelson et al. 2016), the educational context for teaching new technologies (Koc 2013), how higher education is shaped (Franz and Feld 2015), how women recall and verbalise traumatic child birth experiences (Beck 2016), the build-up to democratic elections in Turkey (Hamarat 2016), the message structure of the tourism market in Ghana (Adu-Ampong 2016), or how educational science students communicate preconceptions regarding teaching and learning (Saban et al. 2007).

The use of metaphors by the media and media advertising is particularly relevant because when a specific structural metaphor is favoured two things occur simultaneously. Firstly, the generalised use of that specific form of understanding promotes the salience of specific aspects of the reality while hiding others that are inconsistent with that metaphor. Secondly, alternative metaphorical conceptualisations, which often co-occur, become less normative. Here, Lakoff and Johnson do not see metaphor construction as an innocent process but as an inescapable bias in which inexorably ‘the most fundamental values in a culture will be coherent with the metaphorical structure of the most fundamental concepts in the culture’ (1980, p. 22).

Structural metaphors in CMT can be understood coherently and as a unit due to the metaphorical entailments that connect source and target domains in a process termed ‘mapping’ (Kövecses 2005). The term ‘entailment’, which is used in linguistics in a completely different context, acquires a different connotation in Lakoff’s mind: ‘a conceptual metaphor […] can carry over details of that knowledge from the source domain to the target domain. We will refer to such carryovers as metaphorical entailments. Such entailments are part of our conceptual system. They constitute elaborations of conceptual metaphors’ (Lakoff 1987, p. 384). The entailments are essential to intuitively understanding any implication that metaphors carry within. For instance, by describing paying taxes in terms of a robbery, listeners can re-construct in their minds many entailments of the robbery schema—the good guys and the bad guys, the inherent violence, the injustice of it, and above all, it also implies a course of action (i.e., paying taxes should be avoided). The power of entailments in CMT is that they do not require to be made explicit. In the example above, the consequence that taxes should not be paid is inferred without the necessity of explicitly verbalizing it. People can reach that conclusion by themselves as long as they share a common understanding of what a robbery means in our culture.

Structural metaphors in advertising are effective because they are simple ways of communicating complex attributes and associations. The entailments of a specific metaphor are not understood separately but as a whole, in a gestalt way, that is, ‘a whole that human beings find more basic than the parts’ (Lakoff and Johnson 1980, p. 70). Such metaphors can organise experience in terms of measures understandable for individuals, like in natural dimensions (parts, stages, causes), and they typically an oversight because they ‘seem to us to be *natural kinds of experiences* [emphasis in the original]’ (Lakoff and Johnson 1980, p. 117). Such property of going undetected is pivotal in the normalisation process promoted by advertising. By drawing on gestalt formulations that people usually are familiar with, advertisers introduce new behaviours or popularise existing ones (e.g. OSB) in terms of experiences that people already understand and, because it is always the case in advertising, value as positive.

## Method

### Data Collection

A sample of OSB television advertisements was selected for the present study (N = 135). The study was conducted in the context of a broader study in which all the authors took part (Lopez-Gonzalez et al. 2017a, b). Advertisements were downloaded from the official *YouTube* channels of 29 different betting brands.^1^ The number of advertisements per brand selected ranged from 1 to 19. These brands were selected based on their popularity on specialised internet forums, profit rankings in the gambling trade journal *iGaming Business*, and their presence as sponsors, official partners, or regular advertisers in sport events. The sample comprised advertisements that met the following inclusion criteria:

(1) only those advertising soccer were included, thus excluding horse and dog racing as well as other sports whose popularity is more country-specific; (2) only advertisements from Spain and the UK were selected because these were the two languages that the authors could understand as native speakers, and the representativeness of La Liga and Premier League competitions in European soccer (1st and 3rd, respectively, in UEFA ranking); (3) the advertisements belonged to companies with a legal license to operate in one or both of the countries analysed; (4) the upload date of the adverts had to be from June 2014 to September 2016; (5) the adverts had to have a televisual and temporal format (between 20 and 60 s duration). This latter inclusion criterion excluded made-for-internet promotions that typically allow informal shooting or discussion-like videos including tipsters sponsoring a brand. Also, longer advertisements were excluded because they were unlikely to have been shown on television; (6) the advertisements had to mention OSB, excluding those only addressing offline betting.

Additionally, nine UEFA Champions League, Premier League, and La Liga soccer matches (including pre- and post-match advert breaks) were recorded from May to June 2016. The aim was to double check if those advertisements seen on televised matches corresponded, and were also available, on the brands’ *YouTube* channels, in order to ascertain how similar the two samples were. The results confirmed that every television advertisement was made accessible, sometimes with a few weeks’ delay, via the operator’s *YouTube* profile. Also, the electronic banners around the soccer field were analysed to make sure no betting brand being promoted was excluded from the study sample.

### Procedure

Advertisements were collected and analysed initially by first author over a period of 8 months. To reduce the subjective interpretation in the coding, a sub-sample of 23 advertisements (17% of the sample of 135 advertisements) from British television was randomly selected in order to be coded by all the authors. After discussing the operational definitions regarding CMT, each author independently analysed the selected sub-sample and shared with others his understanding of what constituted a structural metaphor in OSB. After a more nuanced agreement was reached, a second round of analysis was conducted. As three coders were involved in the task, the inter-coder reliability was calculated using ReCal3, an online software designed for nominal data coding designs with three or more coders for which Cronbach’s alpha is not appropriate. The mean inter-coder reliability using Krippendorff’s alpha was 0.956 (SD 0.06, range from 0.78 to 1), much higher than the conservative 0.80 coefficient typically recommended by the author in the context of content analysis (Krippendorff 2013). Once this coding method was deemed appropriate, the first author concluded the analysis of the remaining sample.

CMT was applied in search for entailments that marked the transference of meaning from a source to a target domain. As the purpose of the sample was not being representative, betting brands were not singled out in the analysis and all the advertisements were considered as a single unit of analysis in which the different structural metaphors, to be truly regarded structural as such, should emerge across a variety of betting brands and time periods. The rationale for this was to identify underlying metaphorical conceptualisations as ingrained in the OSB culture of advertising as possible, even as to go unnoticed by advertisers themselves.

A preliminary list of metaphors was identified by the first author. After closer examination, most of those metaphors were discarded based on three criteria. Firstly, despite the purpose of the sample being analytical, a more general representativeness was sought after in the metaphors selected. Thus, metaphors identified in less than one-fifth of the advertisements were excluded. Secondly, the selected metaphors needed to be *structural*, with a detectable source domain that systematically and consistently framed the domain of significance of sports betting. Thirdly, the selected structural metaphors and their entailments were better positioned in connection with the ongoing research conversation regarding gambling advertising and had more far-reaching implications in terms of socially responsible gambling.

## Results

The initial coding yielded a preliminary list of 20 structural metaphors, as shown in Table 1. These metaphors appeared as rarely as once and as frequently as 74 times.

**Table 1:** List of preliminary structural metaphors identified in the initial coding

Rank	Metaphor	Frequency (%)	Rank	Metaphor	Frequency (%)
1	Sport	74 (54.8)	11	Soap opera	2 (1.4)
2	Market	48 (35.5)	12	Officiating a game	2 (1.4)
3	Act of love	29 (21.4)	13	Having an argument	2 (1.4)
4	Natural environment	27 (20)	14	Cooking	1 (0.7)
5	Shared language	6 (4.4)	15	Giving birth	1 (0.7)
6	Conquest	5 (3.7)	16	Creating a work of art	1 (0.7)
7	Luxury item	4 (2.9)	17	Learning	1 (0.7)
8	Robbery	3 (2.2)	18	Chatting with friends	1 (0.7)
9	Military discipline	3 (2.2)	19	Driving	1 (0.7)
10	Planet discovery	2 (1.4)	20	Awakening	1 (0.7)

It was determined that only four of those metaphors met the inclusion criteria in the way that they could be considered common reiterative metaphorical constructions in which OSB was structurally presented in terms of another source domain. The identified metaphors constructed the activity of betting were in terms of (1) an act of love, (2) a market, (3) a natural environment, and (4) a sport. In this section the whole mapping for each metaphor is described, followed by a discussion of the implications for understanding OSB advertising in the relation to the metaphors identified.

### Betting is Loving

In the love metaphor, sports betting is understood in terms of an act of love. Table 2 summarizes the entailments from the love domain borrowed by OSB as seen in its advertisements. In this metaphor, the motivation for betting on sports transcends the mundane search for easy money or fun and is construed according to a deeper sense of meaning. Betting is neither presented as a rational form of money expenditure, nor as a frivolous consequence-free entertainment. Instead, the love domain conveys the notion that betting is rooted deeper in the relationship between sport and fans.

**Table 2:** Betting (target domain) is an act of love (source domain) metaphor

Love	Betting
Love relationship	
Love for another human being	Love for a team
Being loyal to someone	Betting on your team
Cheating on someone	Not betting on your team
Being loved in return	Winning money
Dull relationship	Following sport without betting
Sex	
Physiological manifestations	Emotion of betting
Orgasm	Won bet
Friend relationship	
Loving friends	Betting with friends
Giving good advice to friends	Giving betting tips to friends

The core concept of Betting is Loving is that placing a bet on your team is equivalent to showing your love for whatever that team represents: a city, a nation, or simply the game of football as a whole. Complementarily, betting against an enemy (*hate*-*betting*) could also be interpreted as an act of love and loyalty. A quintessential example of this is the encouragement of Scottish soccer fans (Scotland having not qualified for the Euro 2016 soccer tournament) to bet against England in the tournament. Betting can be conceived then as supporting or backing the preferred team. Following the metaphor, a lack of support would be cheating, as it is generally understood in a love relationship, whereas to continue betting would mean to be loyal and also to have trust in the positive outcome of that relationship/game. An entailment derived from understanding betting in terms of loving is the implication of what bettors can expect to gain from betting. A reciprocal act of love finds love in return whereas non-corresponding love ends up empty handed. Similarly, the characterisation of teams or athletes as loved ones prevents bettors from stopping betting on them, even when they are losing more than they are winning (i.e., *in sickness and in health*).

One of the fundamental experiences inherent to love is the sexual encounter. Inherent to sex are the attributes of immediacy, impulsivity, sensitivity, sensorial alert, as well as joy. Some of the advertisements that make use of the love metaphor portray live betting as being an equivalent of sex, showing, for instance, what appears to be an enhanced skin conductance and accelerated heartbeat. The tension and anticipation derived from the game is blended into the one from the bet, making it difficult to distinguish one from the other. Goal celebrations, profusely depicted in betting advertising although not always with the love metaphorical meaning, work both as figurative orgasms as well as visual manifestations of won bets (which are otherwise typically hard to visually represent). Similarly, a game without betting is conceived as a dull, non-satisfactory relationship that lacks the excitement of a lively experience, as illustrated by mottos like ‘making sport more exciting’, ‘bringing the game to life’, or ‘turning an ordinary game into an extraordinary one’.

This metaphorical construction is especially efficient in playing down the bettor/bookmaker relationship that defines betting, while emphasizing the bettor/team relationship. In an attempt to circumvent the peril of betting being perceived as a monetary wall that dampens the emotional bond between fans and football, the ‘betting is loving’ metaphor helps to reinstate the original fan connection with the game as demonstrated in phrases like ‘there are fans and there are ultimate fans’ by which true hard-core fans need to become bettors in order to express their fanship. As such, betting works as a facilitator of the fan identity, as exemplified in phrases such as ‘you live and breathe [ambiguously, sport or betting]’, or ‘for the love of the game’.

Beyond romantic love, betting can also be understood as love in terms of a friendship. True friendship and the associated camaraderie are long-lasting bonds that need to be nurtured and taken care of. By betting along with friends, such bonds are strengthened because friends live new experiences together and build a shared memory. Also, those who know more about sports share their love within the group by offering tips that could help their friends win some money. Some advertisements depict friends encouraging each other to bet and advising them on the most probable outcomes of a game.

### Betting is a Market

The market metaphor is widely used by betting brands. In this metaphor, OSB is represented as a stock market wherein bets are products that bettors buy. Furthermore, sport competitions are the real world where events happen and cause stock to change its value, and bettors try to interact with it, observing events in sports, and predicting how team performance will impact bet value. Table 3 highlights the main components of the source domain that are mapped in the target domain to make betting understandable in those terms.

**Table 3:** Betting (target domain) is a market (source domain) metaphor

Market	Betting
Market	
Rational	Predictable
Supervised by authorities	Legal and regulated
Fair trade	Fair odds
Broker	
Professional, expert	Expert bettor, knowledgeable fan
Trend analysis	Sport data and statistics analysis
Intermediary	Bookmaker
Competition	Other bettors
Functioning	
Stock price fluctuation	Bet value change
Missing purchase	Missing bet
Selling stock	Cashing out
Insider trading	Tipping

Many adverts naturally use the expression ‘betting market(s)’ as a neutral form of referring to the activity of gambling on sports. Understanding betting as a market entails that sport events are rational, well-regulated and overseen, and that the outcome of a bet is the rational result of a number of interacting variables, that with enough analysis and knowledge, can be successfully predicted. The underlying meaning derived from the notion of market is that betting is a financial domain defined by making money (or limiting the loss of it). In such portrayals, bettors can be decoded as buyers, brokers or investors, and tipsters as financial advisors. The task of bookmakers is to serve as intermediaries between the games and the betting market, offering the best possible conditions (odds, money back, guarantees, etc.) to navigate the market and make the biggest possible profit from it. As in any market economy, knowledge and analysis are essential against a competitive backdrop. Rival bettors might outsmart others, as it is in the case of advertisements promoting exchange betting.

Betting platforms as shown in OSB advertisements often resemble the stereotypical image of stock market boards, with dynamic visualisation of price changes. As in the stock market, a fast-changing reality needs an instantly updated platform to catch up with the speed of modern world (as exemplified in the adverts that state ‘I can see everything the very second it happens’, ‘’I can be in a hundred stadiums… all at the same time’). Some adverts include elements such as equations, statistics, or other undetermined visual elements that largely evoke the mathematical basis of OSB and an outcome based on professional skills. A character in one particular advertisement is specifically referred to as ‘the professor’ but other examples include exotic narratives in which characters calculate the odds of a striker scoring a header over a defender whose eyes will get irritated because of his hair wax, in turn predicted by a newly signed sponsorship deal with a hair wax company. Animation utilizing techniques such as stop motion or frozen images help to communicate the idea of games as events that need to be paused in order to be meticulously analysed.

In-play betting (according to some OSB advertising) requires the same attention as the stock market as prices vary constantly. This is why some of the adverts that utilize this metaphor advise bettors to stay focused not to miss any betting opportunity that might occur during a game. In this context, the market metaphor is particularly useful to introduce a new concept: cash out. Cashing out per se is already a metaphor extracted from the source domain of poker. When a gambler does not want to play anymore, he or she can convert the chips back into money. However, in OSB, cashing out is understood as a way to adapt to the changing cycles of the market. By cashing out, bettors minimise their losses from downward trend bets, as when selling stock, as illustrated by a character in one advert saying ‘when the moment is right, take your profit’. The combination of speed allowed by the platform and the cash out functionality offer a greater opportunity of being successful in the market as illustrated by a fighter pilot in an OSB advert: ‘In life, you ought to think fast and act faster. That fraction of a second can make all the difference. It could be all or nothing. […] Since my bet is in that danger zone, I can get out, quick’.

A smaller number of advertisements also create situations wherein tips are presented in the form of insider information. In one advert, a young photographer on his first job covering a live football game asks his fellow more expert photographers if they got any tips (about photography). Their response is for him to check out some great bet offers on his mobile phone. If betting is a market, then those closer to that market (like the professionals working in the field) can leak high quality information to outsiders. This mental configuration is visible in the selling proposition of some brands. For instance, betting brands stemming from sport media capitalise on their supposed inside knowledge of the game or their long history of sport coverage. Similarly, betting brands from networks holding broadcasting rights present themselves as a more viable option to predict the outcome, reinforced by the pre-game analysis and the expert commentary during the games, and typified in OSB advert claims such as ‘more in the know’, or ‘more insight’.

### Betting is a Natural Environment

In the ‘nature metaphor’, betting is associated with a natural state in which complex cultural developments are reversed into a more primitive setting. The metaphor implies the understanding of OSB in terms of a natural environment characterised by natural and simple actions conducted by animals trying to survive. Table 4 highlights the main entailments inferred from the mapping of betting as happening in a natural environment. Following this metaphor, the smartest bettors are the ones who succeed in betting, and is the same as the principle of the ‘survival of the fittest’ in the natural context. In risk-laden environments like betting and nature, betting operators offer bettors tools to minimise the uncertainty and turn the environment into a safer environment. Functionalities such ‘edit your bets’ allow bettors to adapt more favourably to the ever-changing reality of live games. However, natural disasters are prone to happen and not usually foreseeable. While bets won are is used as evidence by bettors that they had a natural ability to understand reality and adapt, lost bets are naturalised as external events, out of the bettor’s control. Examples such as ‘when a leg of your accumulator *lets* you *down’*, or ‘What a *shocker* of a late goal, your acca [accumulator] was in great shape’ illustrate how lost bets are understood in terms of external phenomena that bettors had no responsibility in failing to predict.

**Table 4:** Betting (target domain) is a natural environment (source domain) metaphor

Nature	Betting
Survival	
Natural selection	Competition, winning of the smartest
Adaptation mechanism	Functionality to change the bet
Evolutionary advantage	Live betting tools, platform
Act of nature (e.g. bad storm)	Unexpected turn in the game
Population	
Predator	Bettor
Prey	Bookmaker/other bettors
Other species	Other bettors
Animal behaviour	
Natural instinct	Intuition, superstition
Escape, run	Cash out
Alert	Attention
Ferocity	Fast decision-making, reaction
Fear	Anxiety, emotion

Consequently, bettors are seen as predators that compete against other predators for prey. In some adverts, prey can be interpreted to be bookmakers, to whom bettors naturally oppose and want to beat. In other advertisements, such as those promoting exchange betting, other bettors play the role of prey. In this context, some adverts build on a bravery versus cowardice dichotomy to encourage betting to prove one’s skills and knowledge over another bettor or bookmaker, as seen in one advertisement where a character representing the position of the bookmaker says to those watching the advert: ‘In your betting, when you lose, we win, and when you win… well, we still win. Because odds are, you don’t have what it takes to beat us in the long run. But, go ahead, prove us wrong’.

Intuition is the equivalent of instinct, a natural mechanism to predict future events. In reality, very few advertisements make explicit claims about the intuitive components of OSB. Rare verbal examples include to ‘back your instincts’ by betting, ‘follow your heart’, or, probably the most notorious example, a young man who tries to convince his male friends to place a strange bet by saying: ‘I have a hunch, all in! Trust me on this, guys, I have a hunch, all in!’. However, in general intuition is implied in a more nuanced fashion, particularly in relation to intuitive knowledge concerning sport and its potential benefit to predict sporting outcomes.

The ‘cash out’ option, widely promoted in many OSB advertisements, works as an escape route once nature turns into a hostile environment. Some adverts explicitly use the word ‘run’, like in the sentence ‘because sometimes you just gotta take the money and run’, to make sense of what cash out means. It should be noted that the sample comprised advertisements from 2014 to 2016, and cashing out was in many cases a recently added product that needed a proper introduction for those not familiar with it. The conceptualisation of cashing out as escaping offers a sense of security to bettors, implying that bad decisions can be later corrected by simply running.

The natural environment is explicitly depicted in one series of advertisements where a deer tries to escape from a cheetah. Hunting an animal might have also some similarities with playing soccer, a situation that features footballers preying on the ball or tackling the legs of a rival. Bettors are regularly depicted as anxious about the result of a game, and verbal claims in such adverts often repeat the importance of focusing and maintaining the attention of the action, as exemplified by expression such as ‘keeping your eye on the ball’. An alternative metaphorical use of the prey versus predator metaphor would be to consider the bettor as the hunter and the bet as the prey. This interpretation appears reasonable given that some advertisements employ wordings similar to ‘track your live bets’, by which platforms and mobile devices are understood in terms of hunting tools that allow bettors to track their (hopefully badly injured) prey.

### Betting is Sport

The metaphor of betting as a sport is probably the most enduring and transversal in OSB advertising. By using this construction, betting is understood in terms of a sport competition in which bettors become active players rather than passive observers of external events. Table 5 summarizes the mapping of sport entailments in the betting domain. Given the focus of OSB is sport itself, the metaphor is both confusing and persuasive. The portrayal of sport action (e.g., featuring footballers playing) feels natural on a message advertising the possibility of betting on such actions. Whereas the metaphors of nature, love, or market appear to be *constructed*, the association of sports betting and sport feels natural and not human-mediated. However, on the contrary, the metaphor expands the original understanding of betting as gambling *on* sport, and transforms it into gambling *as* sport. The confusion between the content of the bet and the activity of betting per se disguises the human role in the construction of the metaphor, naturalising its understanding in those terms.

**Table 5:** Betting (target domain) is a sport (source domain) metaphor

Sport	Betting
Elements	
Manager	Bettor
Player	Bettor
Playing field	Platform or device
Body	Hand, finger
Game	
Training for a game	Studying
Scouting	Navigating the odds
Playing a game	In-play betting
Champion	Winning bettor
Attending a game	In-play betting
Game strategy	Bet selection
Game action	
Goal	Won bet
Hitting goal post	Near miss
Control over the game	Platform customisation

The main implication of this metaphor for bettors is to see their role activated, fundamentally in two distinct forms: player and manager. Some advertisements explicitly depict bettors playing soccer, running with their mobile phones or dribbling while eating popcorn. Others transform bettors into head coaches managing the game live, describing OSB in terms such as ‘it’s like being there, standing on the side of the field’. One betting company presented OSB with the motto: ‘don’t celebrate sport stars, beat them’. This characterisation is not intelligible unless betting as a sport metaphor is previously shared by the target audience. If the metaphor is accepted, bettors can behave like sportspeople and actively challenge opponents.

Some advertisements play with the idea of blurring the lines between the real sport event and the bet. Consider the use of the word ‘you’ in the following expressions from two different betting companies: ‘you’re in the game and the game is in *you’*, and ‘take control of *your* game’. Both expressions appear to leave open to interpretation which game your game might be. The sports event? Your team’s game? The game bet as it is being developed in your device? If the bettor assumes that, by virtue of the real-world sport event being represented in their personal mobile device, that external event now forms part of the individual’s personal experience, then taking control becomes a natural consequence.

Bets occur within the confines of a mobile screen, which is understood as the playing field of soccer. Again, if the transition from stadium to mobile device is accepted, bettors understand that games are not entirely out of their reach and that actions that have consequences on those games can be performed within the confines of the screen. Consistent with this characterisation is the representation of bettors’ hands (and particularly index fingers) as the executive tool to bet/play. Explicit illustrations include claims such as ‘in my hand I have every tool I need to live bet…’ or ‘if you have a finger, you got everything you need to win’. Sometimes, hands and fingers facilitate the claim that OSB is uncomplicated, with characters using their toes to bet or betting without getting the phone out of a pocket. By doing so, OSB is no longer solely a mental action that occurs inside the brain but a bodily activity that requires a physically active individual.

Another implication of betting being understood as a sport is that, as in any sport, betting can be mastered through preparation, training, and practice. Bettors become strategists that plan games, navigate the odds looking for weak spots, in the same way as scouts look for hidden talents in low profile players. Advertisements accentuate the need for being ready (e.g., ‘I am ready, I am always ready’), of being attentive, and keeping one’s eye on the ball at all time. If the studying of the game is thorough, bettors can become high-performing athletes, as illustrated by an advert in which a bettor is asked by a journalist: ‘An amazing career: 5 league titles [Spanish Liga], 3 Champions Leagues, 2 NBA rings, 2 F1 championships… what else now?’.

Across many of the adverts, my-team-scoring-a-goal-like celebrations can be interpreted either explicitly as such, a goal celebration, or tacitly as a celebration of a winning bet. Often OSB narratives feature in parallel game action and characters supposedly betting on those games, with the inevitable outcome being a celebration (although some adverts cut just before the outcome and only suggesting a positive result). Similarly, advertisements where risk-free types of features are introduced, regularly represent near miss bets with a football hitting a goal post or crossbar, while explaining that even in these situations bettors can recover their initial betting stake.

Bettors being understood as footballers prompts the whole representation of the betting action as a professional soccer match situation. In some adverts, well-known sports commentators narrate bettors’ actions in the same terms as the way a footballer plays, with observations such as ‘that’s a great touch’. In others, the hand movement over the screen is sometimes stop-motioned as replays are offered to appreciate the accuracy of the betting performance. All these elements help to construct the meaning of betting as a sporting activity with all the entailments already associated with elite sport.

## Discussion

The findings of the present study demonstrate that the conceptual metaphor of ‘betting as love’ has implications for betting behaviour. Betting on sports differs from other forms of gambling because many bettors have an emotional connection with their sport teams, athletes, and/or organisations. Arguably, many forms of betting do not occur in the context of cold, rational monetary decisions but are gambled in relation to personal preferences based on identity, belonging, etc. The understanding of OSB in terms of a love act might arguably be deepening such a relationship. Advertisers could be building on the pre-existing sports-fan bonds to introduce their products as part of a love metaphor that, if not followed, bettors could interpret their own behaviour as a failure and being perceived by themselves or others as disloyal. Additionally, the love metaphor does not necessarily entail a romantic love perspective. Male friendship has been proposed in the past as a crucial component in betting advertising narratives (Gordon et al. 2015; Lindsay et al. 2013; Sproston et al. 2015). As in the case of romantic love, this metaphor might lead to an understanding of betting as a form of friendship building, and thus discouraging the ceasing of betting.

Also, the market metaphor is far from ideologically neutral. The market metaphor provides OSB with attributes of professionalism, expertise, and business-oriented activity—attributes that smart people typically use in order to profit financially. Such a metaphorical domain prevents advertisers from explicitly claiming the financial rationale for betting on sports, because it is tacitly inferred by bettors who accept that betting is indeed something that resembles a stock market. Embodied in this metaphor, bettors see themselves as businesspeople who make rational decisions. One of the potential problems with the market metaphor is that it brings to the surface a traditional incoherence of betting behaviour. Researchers who have empirically investigated bettors’ behaviour from a purely financial profit maximisation point of view have failed to explain sports betting. Bettors appear to behave more like consumers, maximising utility obtained from the betting product (e.g., having a good time, ‘buying entertainment’, enhancing ego), than investors (Paul and Weinbach 2010). However, betting marketing is increasingly advertising more complex and exotic bets with higher expected losses (i.e., being more probable that bettors will lose), that are hard to calculate even by experienced bettors, as has been detected in betting marketing campaigns in Australia (Gainsbury and Russell 2015; Hing et al. 2017) and the UK (Newall 2015). Arguably, the business-oriented underlying marketing strategy of bookmakers might be antithetical to the actual hedonistic purpose of most bettors. In other words, in some instances, betting marketing could be addressing bettors as businesspeople while bettors are behaving as fans.

Gambling scholars have frequently proposed normalisation as one of the main and more long-lasting effects of gambling advertising (Gainsbury et al. 2013; Lamont et al. 2011; Parke et al. 2014; Pitt et al. 2016; Thomas et al. 2012b). Normalisation is a long-term process that also includes sub-processes of legal and cultural legitimation (Scott 1995). Understanding OSB in terms of a natural environment could arguably facilitate the legitimation of betting across different cultural societies. By naturalising betting and inherently associating it with sport, future bettors might cease to consider betting as a marketing-fuelled purchase option and regard it as a natural component of sport. This is precisely how Lakoff and Johnson (1980) understood gestalts to work in metaphors, in the sense of natural kinds of experiences that go unnoticed by most people. The natural approach, additionally, might suggest the construction of gambling in terms of an inevitable innate behaviour, as old as humanity itself. The same as instincts and sexual relationship patterns, here, gambling behaviour is part of our DNA, the cultural manifestation of a biological attribute. As such, the love and nature metaphors partially interact, forming a wider natural setting in which betting appears to be mirroring more primitive, and inescapable, behaviours. Market and nature metaphors also conflate the understanding of betting as an inevitable process that escapes individual volition. Also, both metaphors are underpinned by the competitive backdrop in which bettors (the same as sportspeople) need to compete in order to survive/win.

The myth of gambling as a sport has previously been hypothesised (McMullan et al. 2012; Milner et al. 2013). Sport metaphors have been identified in distant contexts such as politics, and permeate the ordinary language of English speaking societies with idioms such as ‘rain check’, ‘front runner’, ‘stepping up to the plate’, ‘giving a heads up’, or ‘out of someone’s league’ (Semino 2008; Semino and Masci 1996). The notion of sport communicates health attributes that most brands want to be associated with: success through work, body consciousness, fat and sugar-free diet and exercise, team-building and cooperation, globally recognisable but still locally relevant commodity, and/or joyfulness and diversion. Other forms of gambling, such as poker, have also been found to try to associate their equity brand with sport attributes by means of sporting celebrity endorsements (Lopez-Gonzalez and Griffiths 2016a). In fact, the sport metaphor could be interpreted as a two-way road. Not only is betting being understood as a sport but, simultaneously, sport is also understood in terms of betting. Recent research with Australian gamblers shows that sport and betting share a symbolic alignment. Sport is being increasingly interpreted through the ‘odds lens’, with plenty of terminology from betting language domain permeating the media coverage of sport (Deans et al. 2017).

The fact that many OSB advertisements employ the ‘betting is a sport’ metaphor, implies the control over the sport events by emphasizing the control over the platform (Lopez-Gonzalez et al. 2017a). This poses a major challenge for policy makers. In a distant but analogous context (such as football fantasy games), experiments manipulating advertisements have demonstrated that participants perceived greater control over their actions under the conditions in which more expert information and bigger customization of the platform were emphasised in the advertising narratives (Kwak et al. 2013). Fantasy sports have had a long dispute with American authorities over its characterisation as games of skill or chance (Rose 2015). Although the skill versus chance dilemma might not be an issue from a legal standpoint for online sports betting (Lopez-Gonzalez and Griffiths 2016b), it might still be relevant from a perception point of view, because potential bettors might be more interested in engaging with products they perceive as active rather than passive.

In addition to identifying a number of narrative metaphors, the present study also observed an absence of traditional gambling advertising narratives. For instance, the dream metaphor, whereby the probability of winning is enhanced by framing the gambling experience as a dream where anything can happen, has traditionally been used in advertising (Binde 2007; Sklar and Derevensky 2010), but was not identified in the studied sample of adverts in the present study. One interpretation could be that modern OSB advertising does not focus on gambles where the probability is minimal (but whose prize is life-changing). In such narratives, the dream metaphor is essential to sell hope for an unlikely event. Conversely, dream metaphors are arguably a poor fit for OSB for two reasons. First, winning on a bet placed is not a rare occurrence. Even problem gamblers win frequently. Second, dreaming is a passive action that individuals cannot control, and therefore it adds little to the construction of betting as an active and potentially skilful activity.

## Conclusion

The present paper has argued that below the most visible framework of online sports betting advertising messages lie metaphors that structure the understanding of betting. The paper has contended that Lakoff and Johnson’s CMT is a robust and reliable theory to assess the deeper meaning of advertising narratives and how bettors might be perceiving those messages. The multiplicity of forms that OSB advertising adopts accentuates the need for a platform-neutral approach that analyses how betting activity is constructed in different settings. In this regard, CMT has helped to identify four underlying metaphors (i.e. love, market, nature, and sport) that systematically, across 29 different betting brands and two countries, shape the understanding of OSB. Gambling companies have a social responsibility in the construction and dissemination of particular ways of understanding sports betting, as well as in the discouragement of alternative forms. It is relevant to understand the way advertising messages might promote specific metaphors for individuals to make sense of a widely accepted activity such as gambling on sport events. In this sense, more research is needed to evaluate how gamblers indeed perceive those messages, and whether they conceptualise betting in their minds the way advertising is envisioned.

## Footnotes

1 The advertisements that were analysed included the following brands: *Betfair, Bet365, William Hill, 888 Sports, Skybet, Titanbet, Marca Apuestas, Sportium, Unibet, BetVictor, Marathonbet, Paf, Interwetten, Paddy Power, Ladbrokes, Coral, Luckia, Betfred, Betway, Betsafe, Bwin, Codere, Bet*-*at-home, 10Bet, Bet Stars, Dafabet, 188Bet, Sun Bets, NetBet.*
